# Carbon Material Optimized Biocathode for Improving Microbial Fuel Cell Performance

**DOI:** 10.3389/fmicb.2016.00006

**Published:** 2016-01-26

**Authors:** Hairti Tursun, Rui Liu, Jing Li, Rashid Abro, Xiaohui Wang, Yanmei Gao, Yuan Li

**Affiliations:** ^1^Beijing Engineering Research Center of Environmental Material for Water Purification, Beijing University of Chemical TechnologyBeijing, China; ^2^Beijing Key Laboratory of Membrane Science and Technology, College of Chemical Engineering, Beijing University of Chemical TechnologyBeijing, China

**Keywords:** microbial fuel cells, biocathode, carbon materials, power generation, coulombic efficiency

## Abstract

To improve the performance of microbial fuel cells (MFCs), the biocathode electrode material of double-chamber was optimized. Alongside the basic carbon fiber brush, three carbon materials namely graphite granules, activated carbon granules (ACG) and activated carbon powder, were added to the cathode-chambers to improve power generation. The result shows that the addition of carbon materials increased the amount of available electroactive microbes on the electrode surface and thus promote oxygen reduction rate, which improved the generation performance of the MFCs. The Output current (external resistance = 1000 Ω) greatly increased after addition of the three carbon materials and maximum power densities in current stable phase increased by 47.4, 166.1, and 33.5%, respectively. Additionally, coulombic efficiencies of the MFC increased by 16.3, 64.3, and 20.1%, respectively. These results show that MFC when optimized with ACG show better power generation, higher chemical oxygen demands removal rate and coulombic efficiency.

## Introduction

Microbial fuel cell (MFC) is an emerging and rapidly developing interdisciplinary technology that combines biotechnology, environmental engineering, and electrochemistry (ElMekawy et al., 2014; Sharma et al., 2014). MFCs use electrochemically active microorganisms as catalysts to convert chemical energy directly into electrical energy and are expected to realize the production of clean energy during sewage treatment (Mohan et al., 2014). Based on thermodynamic theory, taking acetic acid as the electron donor and oxygen as the electron acceptor, the maximum theoretical voltage of a MFC system is 1.105 V (Logan, 2008). Currently, the open circuit voltage achieved by MFCs is almost equal to that of traditional fuel cells. However, the achievable output power is still at a low level. Thus, most studies of MFCs are still stuck in the laboratory stage owing to their inefficiency in large-scale applications. The main factors influencing the electricity generation performance of MFCs include exoelectrogens (Sun et al., 2012; Debuy et al., 2015; Rimboud et al., 2015), reactor structure (Izadi et al., 2015; Tian et al., 2015), electrode material (Guerrini et al., 2015), and substrate type (Zhang et al., 2013; Tang et al., 2014; Zhang et al., 2015a). It is generally believed that the electrode material is one of the most critical factors determining MFC performance.

An excellent electrode material should have qualities such as high conductivity, low corrodibility, high specific surface area and porosity, suitability for microorganism growth, and low cost (Wei et al., 2011b). Because many carbon-based materials such as carbon paper (Zhang et al., 2012), activated carbon (Zhang et al., 2014b; Pasupuleti et al., 2015), carbon cloth (Wang et al., 2013), graphite granules (GG; Ye et al., 2015), and carbon fiber brushes (Lanas and Logan, 2013; Liao et al., 2015) have all of these qualities, nowadays they are widely used as MFC electrodes. There has been a number of works carried out on anode material modification and optimization to obtain maximum output power and to improve MFC electricity generation performance (Liang et al., 2011; Li et al., 2014; Liu et al., 2014; Ge et al., 2015). The results of these studies have shown that such approaches can efficiently shorten the MFC startup time, increase the anode biofilm activity, reduce the resistance and increase the output voltage of the system.

Microbial fuel cell cathodes can be divided into chemical and biological cathodes. To improve their performance, chemical cathodes often require precious metals (Quan et al., 2015), metal complex catalysts (Zhang et al., 2015b), or an electrolytic medium (Wetser et al., 2015) to be involved in the reaction. The high cost of suitable catalysts and the easily caused secondary pollution limits the development of chemical cathodes. In contrast, using functional microorganisms as the catalyst, biocathodes have the advantages of low cost, sustainable operation, and wide application. Zhang et al. (2012) found that the use of graphite felt in biocathodes improved catalytic activity toward the oxygen reduction reaction beyond that achieved with carbon paper and stainless steel mesh. Carbon nanotube (Jourdin et al., 2014) and polyaniline/tourmaline (Zhang et al., 2014a) modified electrode were also found to improve biocathode performance by enhancing bacteria–electrode interaction and microbial extracellular electron transfer. Zhang et al. (2011) compared the performance of three types of electrode materials: graphite brushes, GG, and graphite brushes + GG. They found that the MFC startup time was shorter with the graphite brushes + GG cathode than with graphite brushes alone, and a maximum power density of 38.2 ± 12.6% could achieve a correspondingly higher coulombic efficiency.

All previous studies focus on pre-MFC startup, using different electrode materials, applying processing or modifications to the test materials to observe the resulting impacts on MFC startup time and MFC performance (at its stationary phase). However, the electricity generation performance of an MFC is determined by exoelectrogenic growth, which is sensitive to the external environment. Even if two MFCs started up under exactly the same external environment, their electricity generation performance may still vary. In this study, four MFCs were started up with the same electrode material (carbon fiber brushes). GG, activated carbon granules (ACG) and activated carbon powder (ACP) were added to the cathodes after the MFC output voltage reached the stationary phase. After eliminating the errors caused by different microbial growth situations in different treatment phases, through vertical self-comparison we observed the impact of cathode material optimization on the MFC electricity generation performance and the corresponding effect on the contaminant removal from an entirely new point of view.

## Materials and Methods

### Sludge Inoculation

The inoculation sludge used in this experiment was collected from the mixed sludge of the Beijing Qinghe Wastewater Treatment Plant, China. Part of the sludge was held under anaerobic conditions for 7 days, while the other was held under aerated conditions. 10 mL of each sample (MLSS ≈ 4000 mg/L) were injected into the anode chamber and the cathode chamber of the MFCs.

### Electrode Materials

During the cell start-up phase, both the anode and cathode electrode materials were carbon fiber brushes, which were twisted from carbon fibers and titanium wires (brush head of 3 cm length and 3 cm diameter, titanium wire of 3 cm length). The brushes were soaked in acetone overnight and then heated at 450°C for 30 min in a muﬄe furnace (Feng et al., 2010). After being soaked in HCI and NaOH solution for 18 h in each turn (Kim et al., 2007), the GG (1–5 mm in diameter) and ACG (1–2 mm in diameter) were washed and soaked in deionized water, and then dried for further use. Part of the processed ACG were passed through a 100-mesh sieve to obtain ACP.

### Experimental Apparatus

**FIGURE 1:**
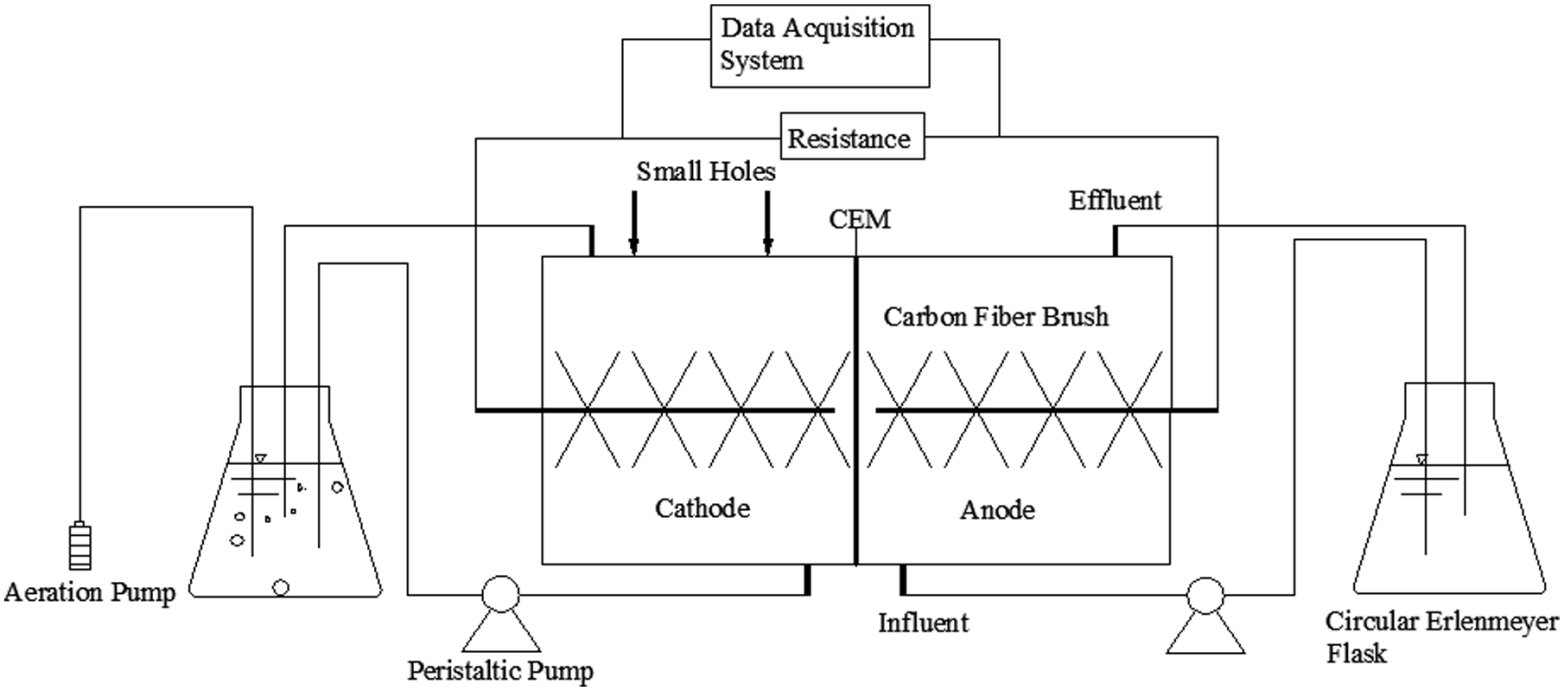
**Schematic diagram of the reactor**.

The MFC reactor in this experiment was constructed of two chambers, the main parts of which were made of plexiglass. The two chambers were both cylindrical, separated by a cation exchange membrane (CMI-7000, Membranes International Inc., USA). Each chamber was 3 cm in length and 4 cm in diameter (net volume of 28 cm^3^), and contained two small holes (1 cm in diameter) on the top usually closed with rubber plugs. The chamber solutions used in the experiments provided nutrients for the electricigens. The anode solution contained 3.4 g/L K_2_HPO_4_, 4.4 g/L KH_2_PO_4_, 1.5 g/L NH_4_Cl, 0.1 g/L MgCl_2_, and 0.1 g/L CaCl_2_, while 1.625 g/L CH_3_COONa was used as a carbon source. The cathode solution contained the same components except for the use of 0.94 g/L NaHCO_3_ (pH = 7, while the phosphate buffer maintained the pH in the range of 7–8 during each batch, to avoid the pH affect the ability to establish an active biofilm on the cathode) as an inorganic carbon source. The anode and cathode solutions were circulated at a rate of 1 mL/min and 5 mL/min using separate peristaltic pumps (BT00-1L, Lange, China) to create an external cycle inside 250 mL circulating containers. An aquarium micro aeration pump was placed inside the circulating container of the cathode chamber to ensure an adequate level of dissolved oxygen. The circulating container was thermostatically heated to 30°C in a water bath to provide a comfortable growth temperature for the microorganisms. A schematic diagram of the experimental apparatus is shown in **Figure 1**.

### Data Acquisition and Analysis

The output voltage data were recorded every minute with a data acquisition card (7660B, ZTIC, China), and its hourly average was archived. The apparent cell internal resistance was measured using the static discharge method (Liang et al., 2007). The voltage values corresponding to the change in external resistance from high to low were recorded, and the corresponding current values were calculated using the following equation

(1) I=U/R

Where, *I* is the output current (A), *U* is the output voltage (V) and *R* is the external resistance (Ω)

Plotting the voltage values versus the current values yielded the polarization curve. The fitted ohmic polarization region of the polarization curve typically showed a linear relationship, the slope of which was the apparent internal resistance. Meanwhile, a saturated calomel electrode was inserted into the cathode chamber as a reference electrode to measure cathode potential. The anode potential was calculated as the cell voltage minus the measured cathode potential. The output power of the cell was calculated using Eq. (2)

(2) $$P=U^{2}/R$$

Where, *P* is the output power (W).

The power density of the cell was calculated based on the area of the cation exchange membrane. Plotting the power density values versus the current values yielded the power-density curve. Generally, the highest point of such a curve is the maximum power density of the cell.

The soluble chemical oxygen demands (COD) of the MFCs were measured according to the standard method. Coulombic efficiency is the ratio between the number of output electrons and the number of electrons that the consumed organic compounds can provide. It describes the energy transfer efficiency of an MFC, and is an important indicator of MFC electricity generation performance. For the present experiments, the coulombic efficiency was calculated as follows:

(3) $$C_{E}=\frac{8Q}{FV\Delta COD}$$

Where, *C*_E_ is the coulombic efficiency (%), *Q* is the total output of the MFC during a cycle (C), *F* is he Faraday constant (96485 C/mol), *V* is the volume of anode solution (mL), Δ*COD* is the change in the COD concentration during a cycle (mg/L) and “8” is the constant when using oxygen as the electron acceptor.

Cyclic voltammetry curve was implemented by electrochemical workstation (CHI-604E, CH Instruments, China) through conventional three electrode system. The morphology of the biofilms on the electrode materials was examined by scanning electron microscopy (SEM; S-3400N, Hitachi, Japan). The samples were processed for imaging according to the method described in Zhang et al.’s (2012) report.

## Results and Discussion

### MFC Start-Up and Stationary Phase

**FIGURE 2:**
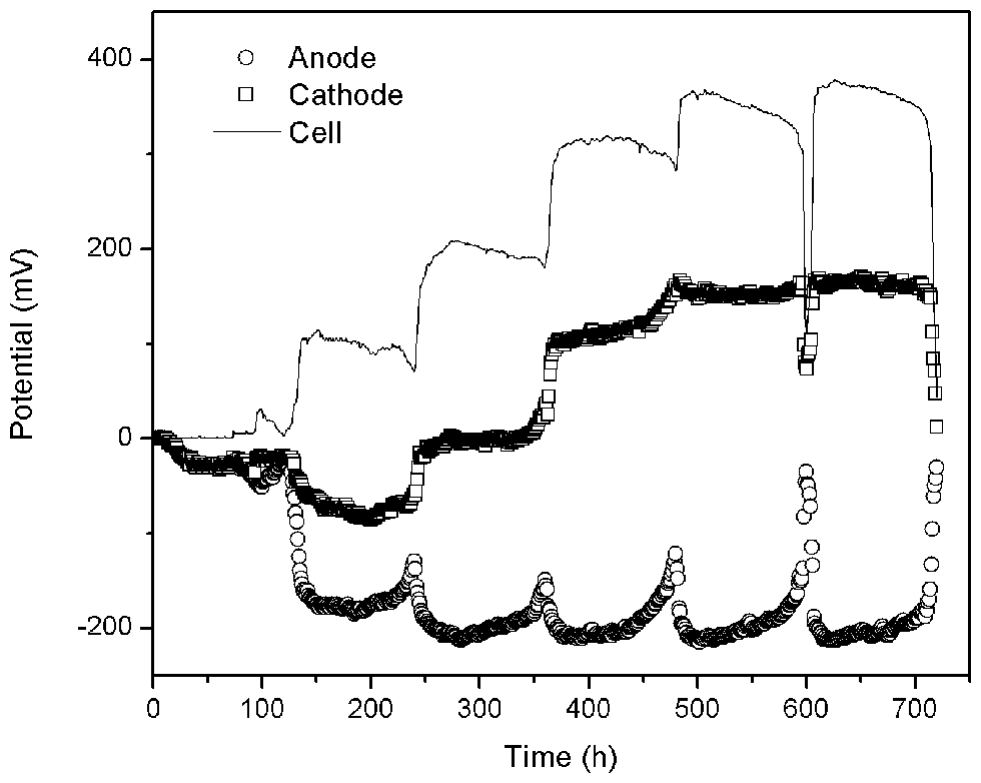
**Electrode potential changes during the start-up period**.

Four reactors were used in this experiment labeled: CFB (control reference), GG (adding graphite granules), ACG (adding activated carbon granules), and ACP (adding activated carbon powder). All four reactors were identical in structure and operating conditions. The cycling time of the solutions was 5 days. During the start-up phase, the generation capacity was low and unstable owing to the lack of biofilm on the electrode material. In the second cycle, anode potential began to drop significantly (see **Figure 2**) due to the rapidly growing of electricigens in the anode chamber. Meanwhile dissolved oxygen without catalyst hardly accepted the electrons form organic matter degradation leading to electron enrichment on the surface of carbon fiber, which caused cathode potential going downward. However, when the biofilm grew-up, cathode potential started to ascend. After 30 days, when the maximum output voltage no longer increased during three consecutive cycles, the MFCs were considered to have successfully started and reached the stationary phase.

The output voltages of the four fully started MFCs ranged between 350 and 385 mV. The corresponding current densities (external resistance = 1000 Ω) were all in the range of 0.50–0.55 mA/cm^2^. These results show that the differences among the four MFCs are unremarkable, and indicate that the microbial growth and distribution of each MFC is relatively consistent and that their electricity generation performance is comparable. As such, these results allowed us to move to the next phase.

### MFC Generation Performance

Thirty hours after the solutions were replaced at stable output voltage; 1 g GG, 1 g ACG and 1 g ACP were added into the corresponding cathode chambers through the small holes at the top of each reactor (**Figure 1**). The earlier results of preliminary experiment suggested that the best dosage of carbon material is 1 g. Therefore, the output voltages were monitored by data acquisition card and the currents of each MFC was calculated accordingly to assess the MFC performances.

**FIGURE 3:**
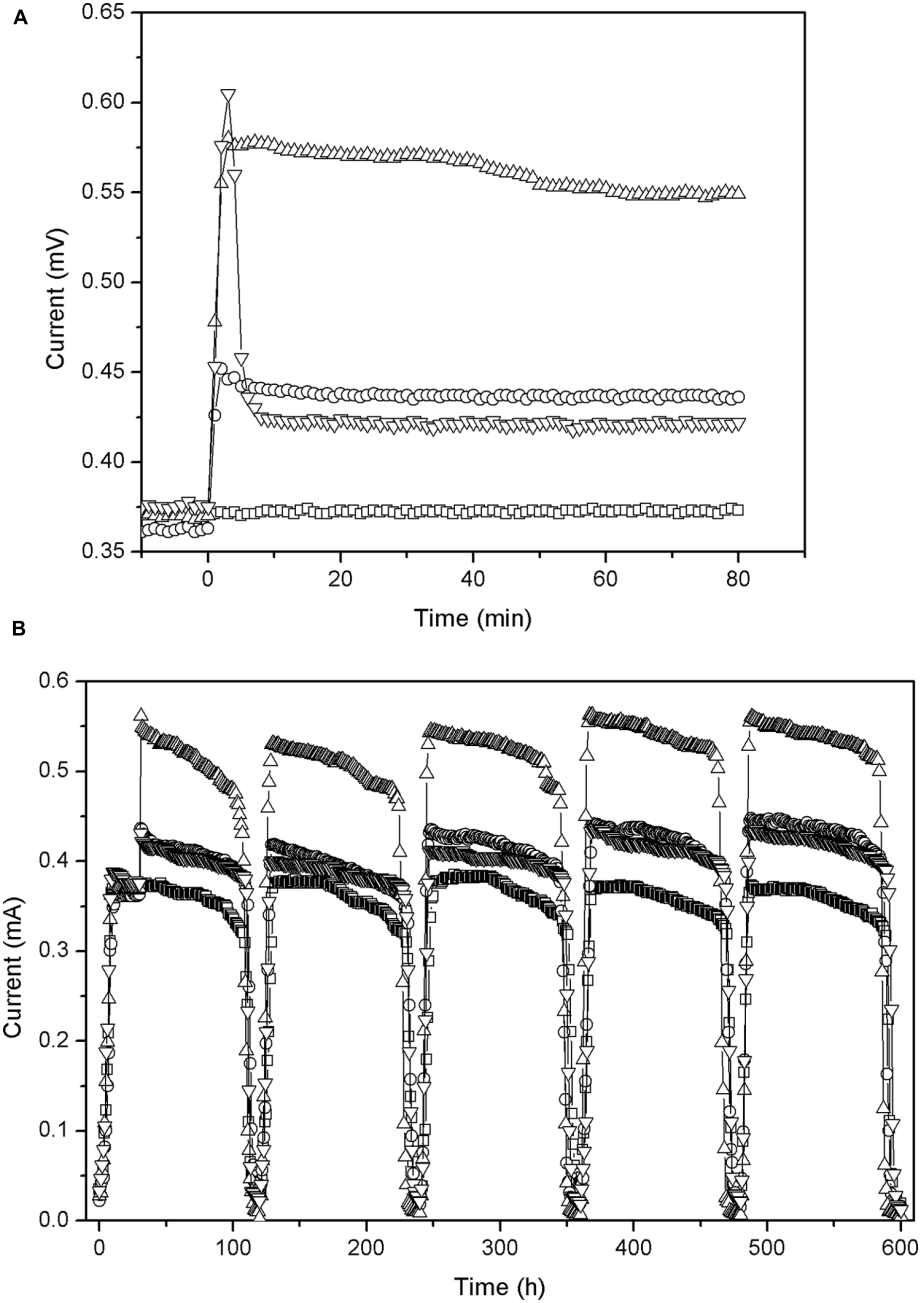
**Change in output current (external resistance = 1000 Ω) before and after addition of carbon material: (A) first 2 h; (B) during five cycles.** -□- CFB, -○- graphite granules (GG), -△- activated carbon granules (ACG), -▽-activated carbon powder (ACP).

As shown in **Figure 3A**, the output currents of all the MFCs (after addition of carbon material) display a significant increase, and their maximum values are achieved within 3 min. This might be due to the following reasons: (1) the electrical conductivity of carbon materials reduced the internal activation resistance of the MFC in a short time. (2) The dry carbon materials that contain oxygen may increase oxygen content of the cathode solutions, thus speeding up oxygen reduction rate and leading to the greatly enhanced cathode performance. (3) Graphite and activated carbon have been reported as effective catalysts for oxygen reduction in cathodes of MFCs (Freguia et al., 2007; Zhang et al., 2014b).

However, after 3 min different MFCs show substantial differences in performance (**Figure 3A**). (1) The current of GG stabilized at around 0.436 mA. (2) The current of ACG continued to increase and reached a maximum 0.575 mA after 10 min while after about 40 min, it started to decline and finally remained at about 0.549 mA. (3) The current of ACP began to drop rapidly, from about 0.605 mA to about 0.422 mA, and then remained stable. At the end of the experiment, when we washed the apparatus, we found that the filaments of the carbon brush blocked the ACG owing to granule’s large diameter. This increased the specific surface area of the electrode to some extent, which may have attracted more aerobic microbes and increased the productivity of ACG accordingly. In contrast, the powder was too small to be blocked by or attached to the brushes effectively, thus passed through the brush filaments into the reactor bottom (the non-conductive dead zone), and part of them passed out of the reactor with the eﬄuent. This resulted in a lower conductivity and underutilized biocompatibility for ACP. Accordingly, after adding the ACP the output voltage and current of ACP increased rapidly and then declined significantly as the powder started to deposit.

**FIGURE 4:**
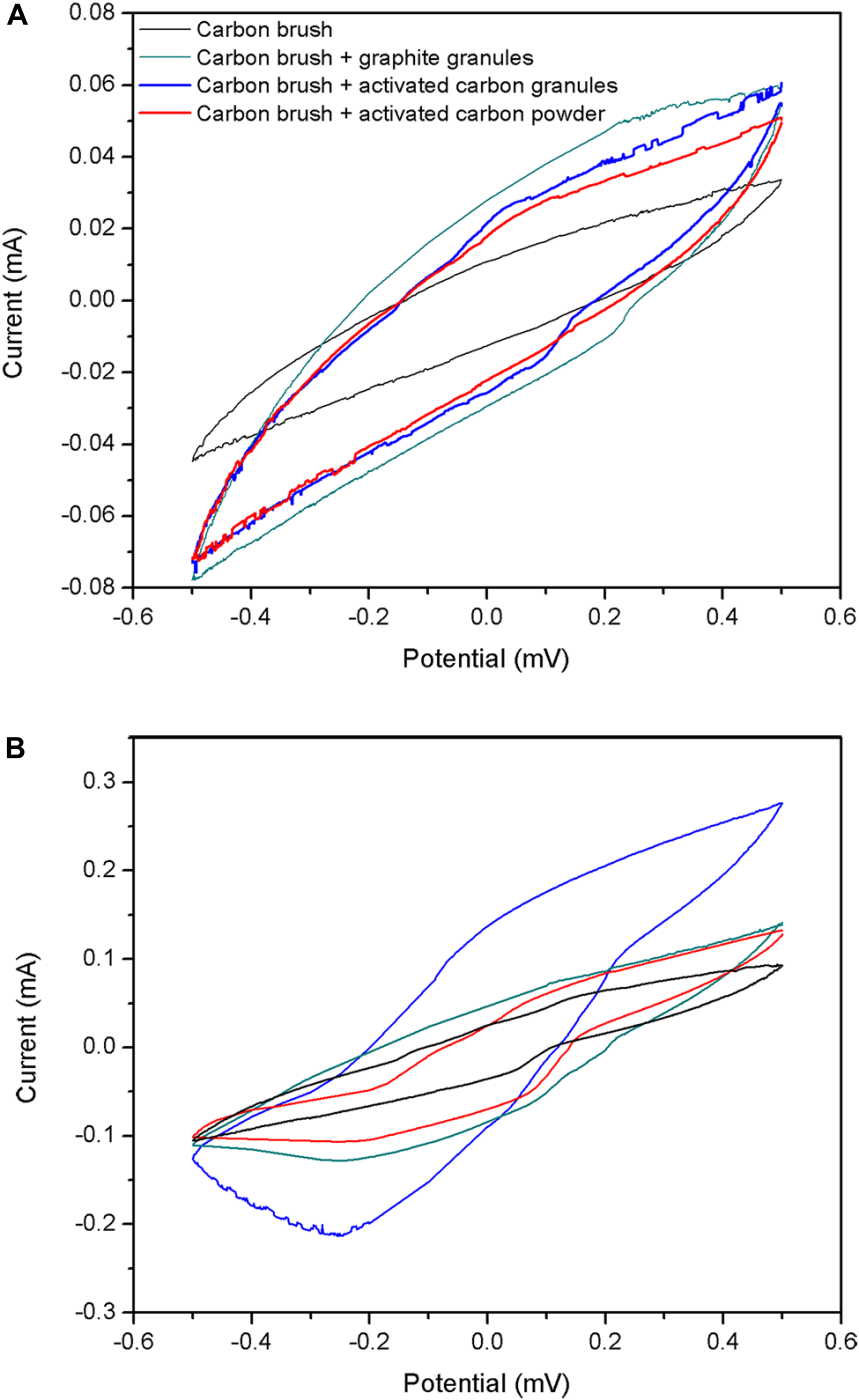
**Cyclic voltammetry curves for cathode electrodes: (A) without biofilm attached; (B) with biofilm attached**.

To investigate the catalytic behaviors caused by chemical catalysis or biological catalysis, four types of electrodes (carbon fiber brush, carbon fiber brush + GG, carbon fiber brush + ACG and carbon fiber brush + ACP) without and with biofilm attached were characterized by cyclic voltammetry. The measurement was performed in air-saturated cathode solution as reported in Section “Experimental Apparatus.” As shown in **Figure 4**, there was no remarkable redox peak in four types of electrodes without biofilm, suggesting that the raw electrodes had no chemical catalysis in this experiment. On the contrary, electrodes with biofilm had remarkable reductive peaks. Moreover, the peak current of carbon fiber brush + ACG electrode with biofilm was higher than that of the others. This result further indicates that electrochemical active microorganisms in the cathodes catalyze oxygen reduction reaction.

**FIGURE 5:**
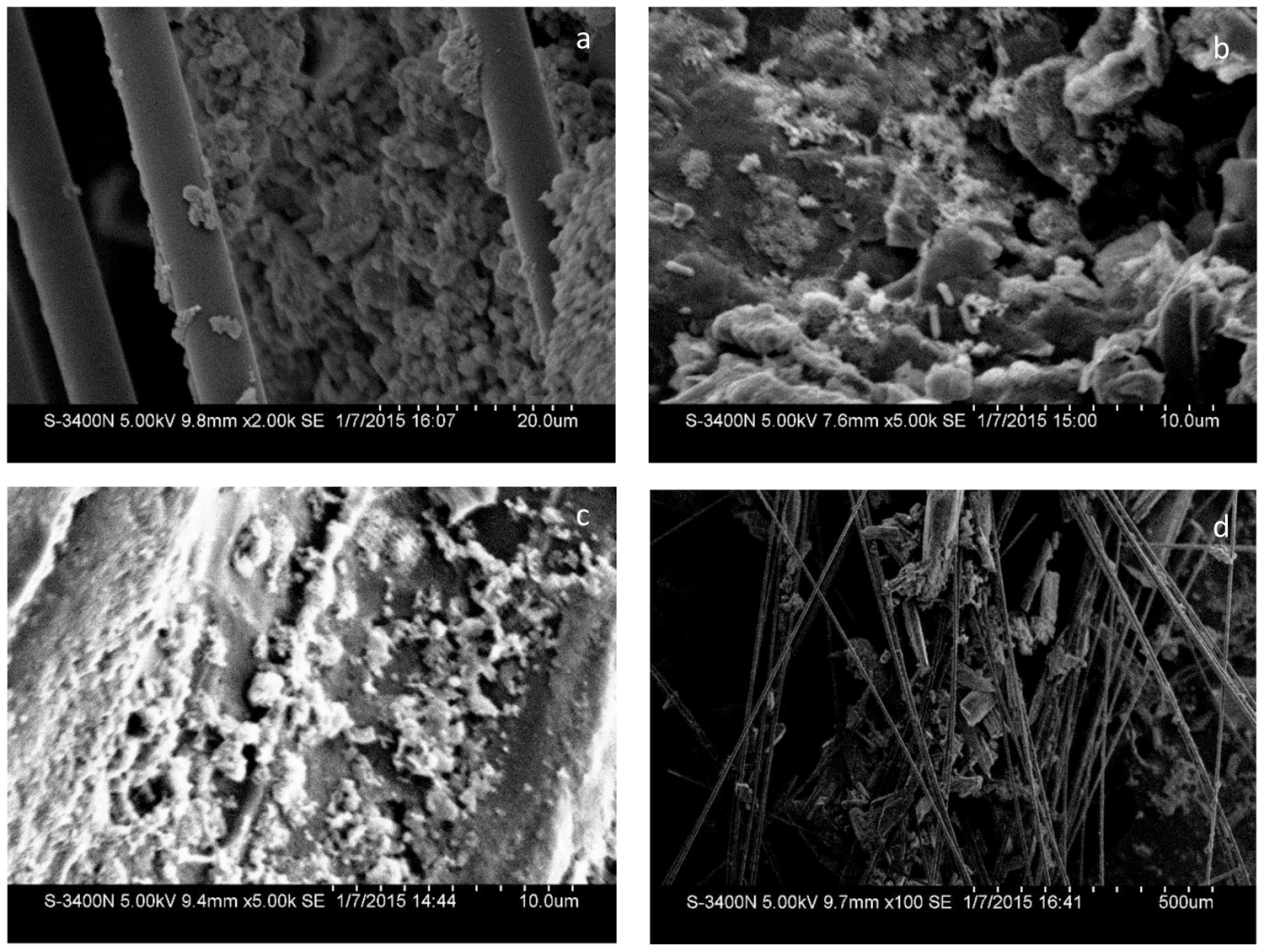
**SEM images of biofilms attached to the cathodes: (a) CFB; (b) GG; (c) ACG; (d) ACP**.

All the MFC reactors remained stable and functional for several cycles after the carbon materials were added (**Figure 3B**). The output current of CFB almost did not change, indicating that the external environment did not influence the power generation of the MFCs during the test period. The output current of the other reactors did not obviously increase beyond the maximum current which was observed just after the addition of the carbon materials. Moreover, the surface morphology of the carbon material observed by SEM (**Figure 5**) showed the biofilm attached on carbon fiber, GG, and activated carbon. Because the carbon fiber surface was smooth, only a small amount of microbes could adhere and most of the microbes clumped together away from the filaments (**Figure 5A**). As a result, the power generation performances of the MFCs were low before the addition of carbon materials. This phenomenon is similar to the observation by Karra et al. (2013) and Sonawane et al. (2014).

The added graphite and activated carbon had large porosity and adsorptive capacity that could spread the clumped microbes adsorbed on the electrode surface and increased the surface area of the electrode. Previous studies suggested that the amount of bacteria on the biocathode should be one of the limiting factors to determine the charge transfer resistance and power generation (Rabaey, 2010; Zhang et al., 2012). Since the area of biofilm in direct contact with dissolved oxygen was increased, the oxygen reduction rate was promoted and the electricity generation performance of the MFC was improved.

In addition, it has been reported that the specific area of activated carbon was approximately 2.4 times higher than the specific area of GG (Wei et al., 2011a). This higher specific area enabled the more effective collection of electrochemical active microorganisms and the performance of ACG increased accordingly.

**FIGURE 6:**
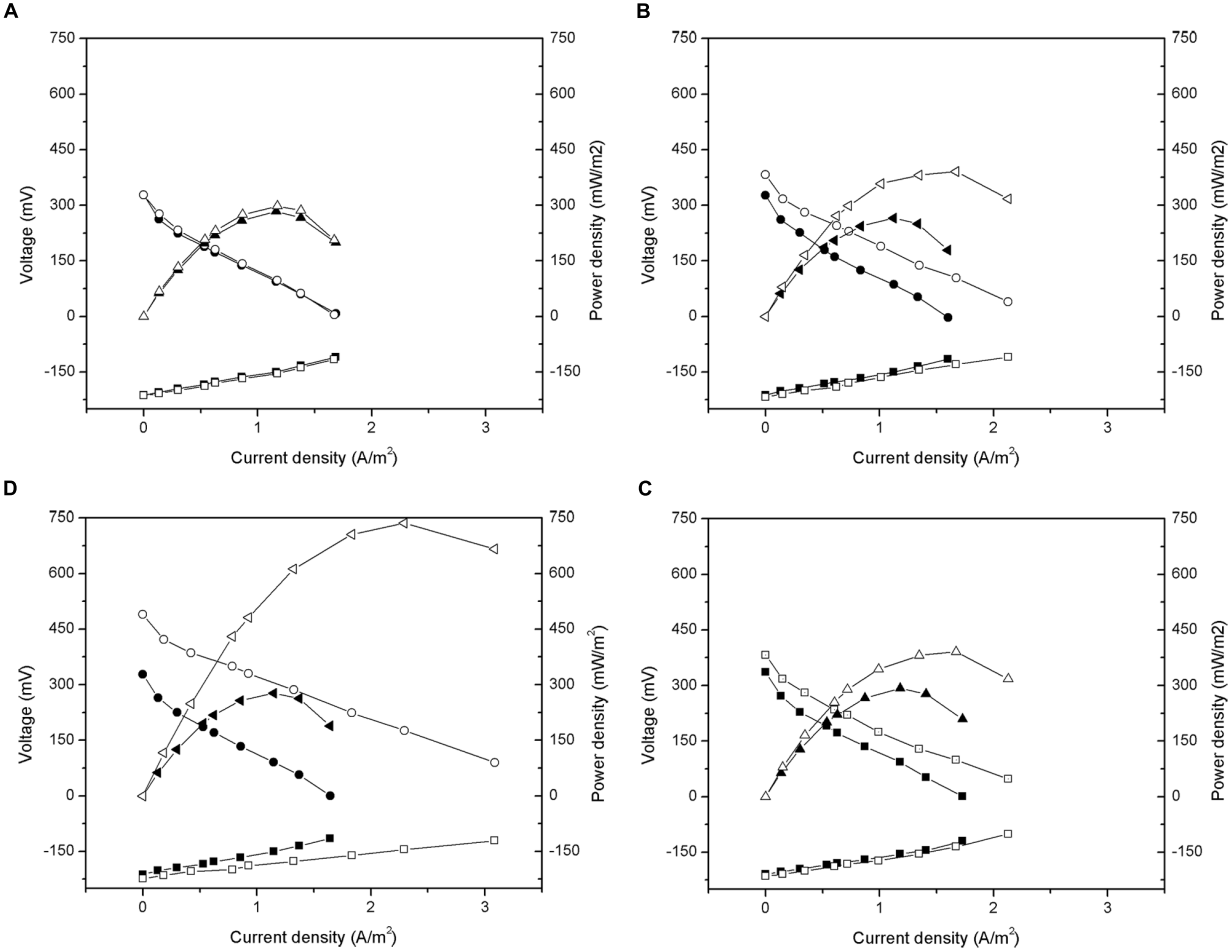
**Polarization and power density curves after addition of activated carbon material: (A) CFB; (B) GG; (C) ACG; (D) ACP.** -■-, -□- Anode potential; -●-, -○- Cathode potential; -▲-, -△- Power density.

As shown in **Figure 6**, the anode potential measured after the addition of carbon material did not have obvious change. Thus, the improvement in the performance of the MFCs occurred mainly because of cathode polarization. After the addition of carbon material, the open circuit voltages of GG, ACG, and ACP were increased by 11.3, 31.7, and 9.5% to approximately 601, 713, and 597 mV, respectively. After linear fitting of the polarization curves, the internal resistances of GG, ACG, and ACP were calculated to be 269, 204, and 299 Ω, respectively, all lower than their initial values. Early research indicated that the total internal resistance of an MFC consists of three components: ohmic resistance, activation resistance, and diffusion resistance (Logan et al., 2006). Ohmic resistance depends on the type of electrolyte and membrane; activation resistance is determined by the activation rate of the electrode surface; diffusion resistance (aka. concentration resistance) is dependent on the diffusion rate of the reaction products transferring toward the electrode surface and solution.

The cathode resistance is generated from the electrochemical oxygen reduction reaction on the cathode surface. Thus, taking the same experimental conditions into account, apparatus, and ohmic resistance, a reduction in cathode activation resistance may have been the main cause of the observed drop in the total internal resistance of all three reactors. This result was similar to Zhang et al. (2011) and Zhang et al. (2014a). Furthermore, the diffusion resistance also decreased owing to the increase in the specific surface area of the electrodes after addition. The in-depth study on the composition and mechanism of change in the MFC internal resistance are still required.

According to equation (2), the output power density of the MFC was proportional to the square of the open circuit voltage, and inversely proportional to its internal resistance. Under the condition that the open circuit voltage increased while the internal resistance decreased, the maximum power densities of GG, ACG, and ACP reached approximately 391, 736, and 391 mW/m^2^, increased by 47.4, 166.1, and 33.5%, respectively, after the addition of carbon materials. This further indicated the significant improvement in the electricity generation performance of the MFCs after the addition of carbon materials (ACG especially) to the cathode. This improvement was caused by the reduction of the internal resistance due to higher specific surface area of the graphite and activated carbon than the carbon fiber brushes. A higher specific surface area could increase the growth of electrochemically active microorganisms, which catalyze the oxygen reduction reaction, and could reduce the activation internal resistance of the cell, thereby increasing the electricity generation performance.

### COD Removal and Coulombic Efficiency

**FIGURE 7:**
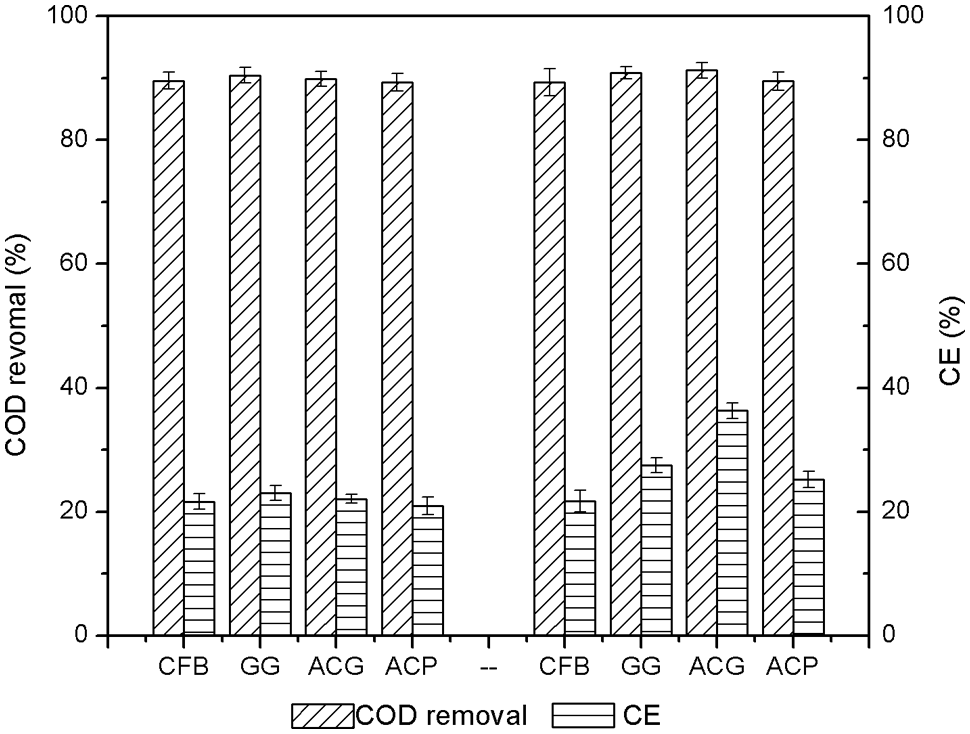
**Changes in chemical oxygen demands (COD) removal and coulombic efficiency before and after addition of activated carbon materials**.

The changes in COD removal and coulombic efficiency before and after the addition of the carbon materials were measured. The COD removal and coulombic efficiency data were collected and averaged from the three cycles before and after the addition of carbon material. The results are illustrated in **Figure 7**.

As shown in **Figure 7**, before the addition of carbon materials to the MFCs, the COD removal rates of the four reactors were between 89.3 and 90.5%. This demonstrated that the microbial growth and the performance of the four anodes were similar. The coulombic efficiency of each reactor was low, only between 20 and 23%. The majority of the energy was lost during the process of converting COD removal to electricity generation. After the addition of ACG, the COD removal increased to 91.2%, indicating that the optimization of the cathode could also have an indirect effect on anode COD removal. Accordingly, after the addition of GG and ACP, the COD removals were almost unchanged but the output power increased a little. This result confirmed that the increased electricity generation performance was mainly determined by the improvement of the cathode. With similar initial COD removals, the coulombic efficiency of GG, ACG, and ACP increased by 16.3, 64.3, and 20.1%, respectively. This further verified that the MFC coulombic efficiency varied directly with the output power density.

## Conclusion

The experimental results of this study showed that the current of an MFC (double-chambers, carbon brush as start-up electrode material) in stationary phase rapidly increased by the addition of carbon materials due to the physical property of the materials. The addition of carbon material increased the specific surface area of electrode material and improved the activity of the catalytic microorganisms toward the oxygen reduction reaction, and thus maintained the high performance of the MFCs in following cycles. As a result, the internal resistance of the MFCs reduced effectively and electricity generation performance improved. Using ACG, the maximum power density increased significantly by 166.1%. The ACG optimized anode also showed higher COD removal rate than those with GG or carbon powder. However, all three-carbon materials improve the coulombic efficiency rate of the MFCs.

## Conflict of Interest Statement

The authors declare that the research was conducted in the absence of any commercial or financial relationships that could be construed as a potential conflict of interest.
